# Gastric Carcinoma: An Unexpected Complication of Loop Gastro-Jejunostomy Done in Childhood

**DOI:** 10.7759/cureus.15900

**Published:** 2021-06-24

**Authors:** Sri Hari Priya Vemulakonda, Rehena Sulthana, Ankit Jain, Abhinaya Reddy, Vishnu Prasad Nelamangala Ramakrishnaiah

**Affiliations:** 1 Surgery, Jawaharlal Institute of Postgraduate Medical Education and Research, Puducherry, IND

**Keywords:** gastric carcinoma, gastro-jejunostomy, biliary reflux, adenocarcinoma, gastrectomy

## Abstract

Gastric adenocarcinoma is the fifth most common cancer worldwide and the third leading cause of death. The major risk factors include Helicobacter pylori infection, genetic factors, environmental factors, and atrophic gastritis. Gastric remnant cancer is gastric carcinoma that develops in the remnant stomach more than five years after distal gastrectomy for benign disease, incidence ranging from 1% to 8%. However, gastric carcinoma after loop gastro-jejunostomy without gastric resection for benign etiology is rare. We report a case of a 45-year-old lady with gastro-jejunostomy without gastric resection done in childhood, presenting with adenocarcinoma at the anastomotic site after 35 years.

## Introduction

Gastric cancer is the fifth most common cancer worldwide and the third leading cause of death [1]. Adenocarcinoma of the stomach is the most common type. Helicobacter pylori and Epstein Barr virus infection, dietary factors like smoked and salted food consumption, environmental factors like poor socio-economic status, atrophic gastritis, and adenomatous polyps are some of the risk factors for developing gastric carcinoma. However, gastric carcinoma after Billroth-II type loop gastro-jejunostomy (GJ) for benign etiology is rare [2]. Here, we report a case of a 45-year-old lady who presented with gastric adenocarcinoma following GJ done in childhood.

## Case presentation

A 45-year-old lady presented to the emergency with multiple episodes of non-bilious vomiting, containing undigested old food particles, for the past two months. The vomitus was associated with epigastric pain, on and off post-prandial abdominal distension, early satiety, decreased appetite, and significant weight loss over the past two months. However, there was no history of hematemesis, melena, or jaundice. The patient gave a history of an upper abdominal surgery in her childhood (around 35 years ago), details of which were not known, and no records were available. At presentation, she had tachycardia (110/min) and was dehydrated. The abdomen was soft with distension of the upper abdomen, and the succussion splash was positive. There was an upper midline vertical scar present, healed with primary intention. Blood investigations showed hemoglobin of 7.7 g/dL, urea of 12 mg/dL, creatinine of 0.28 mg/dL, sodium of 129 mEq/L, and potassium of 4.2 mEq/L.

**Figure 1:**
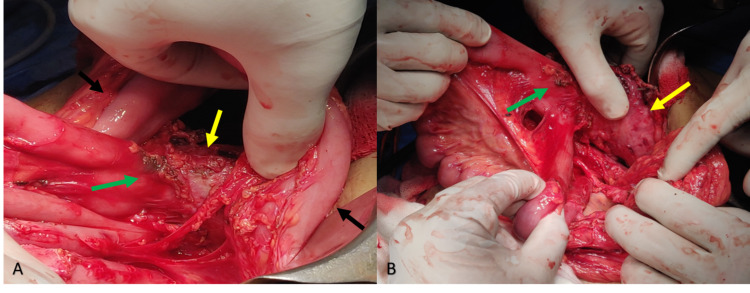
(A, B) Intraoperative images showing the retro-colic gastro-jejunostomy to the posterior wall of the stomach. (Black arrow: Transverse colon. Yellow arrow: Stomach. Green arrow: Gastro-jejunostomy site.)

Upper gastrointestinal endoscopy (UGIE) revealed an ulcero-proliferative circumferential growth involving the lesser curvature, body, and the antro-pyloric region of the stomach. Multiple biopsies taken from the growth were suggestive of gastric adenocarcinoma. Contrast-enhanced computed tomography (CECT) revealed a circumferential growth involving the body and antro-pyloric region of the stomach. The growth was adherent to bowel loops with loss of fat planes with liver and pancreas. She was taken up for staging laparoscopy. Due to the absence of liver or peritoneal metastasis, and no infiltration of the growth into the liver or pancreas, we proceeded with total gastrectomy. However, growth was found adherent to the jejunal loops traversing through the transverse mesocolon. On further dissection, the adherent jejunal loop was found to be retro-colic posterior loop GJ (Figures 1A, 1B).

**Figure 2:**
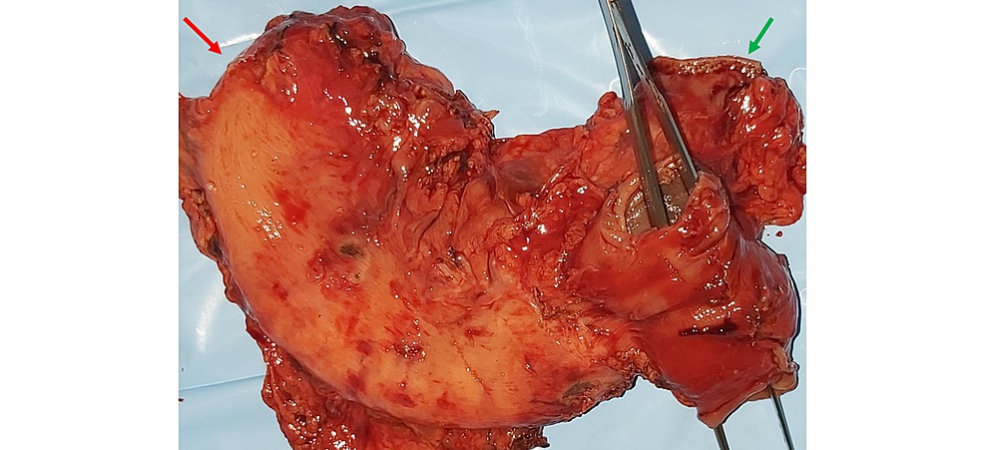
Resected specimen showing the posterior surface of the stomach with forceps passed through the jejunal loop of gastro-jejunostomy. (Red arrow- Stapled esophageal end of the stomach. Green arrow- Stapled duodenal end of the stomach.)

Total gastrectomy with excision of previous GJ loops for a length of 10 cm along the afferent and efferent jejunal loop was done (Figure 2).

Intestinal continuity was restored by Roux-en-Y esophago-jejunostomy and jejuno-jejunostomy. The patient had an uneventful postoperative recovery and was started on capecitabine-based adjuvant chemotherapy.

## Discussion

Except for malignant conditions, loop GJ is commonly done for benign gastric outlet obstruction (GOO) due to peptic stricture, caustic ingestion, inflammatory diseases such as Crohn’s disease or tuberculosis or as part of surgical procedures such as mini gastric bypass bariatric procedure, and pyloric exclusion in complex gastro-duodenal perforations. In the past, distal gastrectomy with loop GJ was used for patients with peptic ulcer disease. Due to the advent of acid inhibitory medications, this surgical procedure is rarely done nowadays. However, it is still done at our center for complications like bleeding ulcers. The most common complication following GJ is stenosis, followed by marginal ulcers and perforation [3]. Gastric remnant cancer (GRC) is the gastric carcinoma that develops in the remnant stomach more than five years after distal gastrectomy for benign disease [4-7]. The incidence of GRC ranges from 1% to 8% [5]. However, gastric carcinoma after GJ without gastric resection for benign disease is rarely reported in the literature [2,8].

Biliary reflux plays a vital role in the pathogenesis of post-GJ malignancy. Bile is the most significant content of reflux, damaging the gastric mucosa, and disrupting the gastric mucosal barrier. In patients with GJ, the gastric mucosa is exposed to continuous biliary reflux leading to alkaline gastritis and intestinal metaplasia, a precancerous condition. This alkaline medium with intestinal metaplasia allows bacterial colonization, which reduces nitrates in the food into nitrosamines which are carcinogenic and proven to cause gastric carcinoma [2]. Given the longer life span of patients with benign disease, such patients with loop GJ are exposed to reflux for a longer time than patients with malignant etiology. With or without gastric resection, the gastrointestinal continuity can be restored by Billroth-II type loop GJ ± Braun’s enterostomy or Roux-en-Y GJ. In addition to the reduced incidence of other complications, Roux-en-Y GJ has been proved to be associated with decreased incidence of reflux and intestinal metaplasia [5-7]. However, the appropriate length of the Roux-en-Y limb, which can completely prevent reflux, is still under debate. Though some argue for a 40-cm loop, the literature supports a loop length of 60 cm to obviate jejunal reflux completely [9].

Patients with loop GJ develop carcinoma commonly at the anastomosis site and rarely in the stomach away from the anastomosis site [5,10]. The reported time interval between loop GJ and gastric carcinoma diagnosis ranges from 15 years to 25 years, the maximum being 50 years [4,8]. The shortest time interval reported is 10 years after surgery for a duodenal ulcer [8]. In our case, carcinoma was diagnosed around 35 years after the previous surgery. The presentation of carcinoma stomach post-GJ would be the same as in patients without prior GJ; pain epigastric region, hematemesis, and GOO are common symptoms. UGIE would be the first investigation for diagnosis. The stomach should be thoroughly examined along with the anastomotic site and GJ's afferent and efferent loops. Adenocarcinoma is the most common histological type of gastric carcinoma arising post-GJ. Intestinal type is more common than diffuse variant in such patients [5]. However, signet-ring cell carcinoma arising from the previous GJ anastomotic site has been reported [4]. High incidence of jejunal mesenteric lymph nodal metastasis and direct invasion into the colon or mesocolon has been noted due to previous surgery and reformation of new lymphatic routes alongside the jejunal stoma [5-8]. A CECT would further help identify the extent of the tumor and its relation with surrounding organs and distant metastatic disease.

In the case of resectable tumors, subtotal or total gastrectomy with gastro-jejunal anastomotic site resection with a wide margin must be performed [5,6,8]. Given the high incidence of metastasis in the jejunal mesentery, en-bloc wide excision of jejunum with mesentery is mandatory [5,7,8]. Despite the extensive local disease, curative or en-bloc resection is possible in close to 90% of cases [5,6,8]. Moreover, many authors have endoscopically treated early GRC successfully with minimum complications [5,6]. None of the cases reported so far received neoadjuvant chemotherapy as the tumors were diagnosed before metastasis, and surgery was the initial treatment given in the cases with gastric carcinoma post-GJ. Therefore, the role of neoadjuvant therapy is not clear. Given the rarity of GRC or post-GJ carcinoma without gastrectomy, the prognosis of these tumors is still debated. However, numerous studies have reported survival similar to patients with similar stage primary gastric carcinoma [5-7].

Though there are no studies available regarding the screening for gastric carcinoma in patients with loop GJ without gastrectomy, annual screening with biopsy of the suspicious region is recommended in patients at risk for GRC [5,6,11]. However, the time period post-GJ for initiation of screening and duration of screening is not clear. Some authors recommend 10 years [11], whereas others recommend that five years interval from surgery is an appropriate time to start endoscopic surveillance [5]. Therefore, a similar recommendation can be made for patients undergoing GJ without gastrectomy for benign pathology, especially given the possibility of endoscopic treatment for early lesions.

## Conclusions

Although rare, gastric malignancy post-GJ for benign etiology can occur. Although these tumors are prone to extensive local disease, curative or en-bloc resection is possible in most cases. Therefore, the survival of these patients is similar to patients with stage-matched primary gastric carcinoma. Routine annual screening with biopsy of the suspicious region is recommended in patients for early diagnosis.
